# Midkine promotes hepatocellular carcinoma metastasis by elevating anoikis resistance of circulating tumor cells

**DOI:** 10.18632/oncotarget.15808

**Published:** 2017-03-01

**Authors:** Bin Sun, Congli Hu, Zhibin Yang, Xiaofeng Zhang, Linlin Zhao, Junye Xiong, Junyong Ma, Lei Chen, Haihua Qian, Xiangji Luo, Lehua Shi, Jun Li, Xianshuo Cheng, Zhengfeng Yin

**Affiliations:** ^1^ Eastern Hepatobiliary Surgery Hospital, Second Military Medical University, Shanghai, China; ^2^ Colorectal Cancer Clinical Research Center, Third Affiliated Hospital, Kunming Medical University, Kunming, China

**Keywords:** midkine, hepatocellular carcinoma, circulating tumor cells, anoikis resistance

## Abstract

Midkine is overexpressed in hepatocellular carcinoma (HCC) and plays a role in tumor progression, but less is known about its role in resistance of circulating tumor cells (CTCs) to anoikis which leading to recurrence and metastasis. The aim of the present study was to analyze whether midkine was associated with HCC progression with anoikis resistance. We found that cultured HCC cells were more resistant to anoikis, which paralleled midkine expression, and midkine treatment significantly inhibited anoikis in a dose-dependent manner. Furthermore, in *in vitro* and *in vivo* assays, knockdown of midkine resulted in significant sensitivity to anoikis, decreased cell survival and significantly decreased tumor occurrence rate. Patients with midkine-elevated HCC had higher CTC counts and less apoptotic CTCs, as well as significantly higher recurrence rate and shorter recurrence-free interval. To understand the molecular mechanism underlying the midkine with HCC progression, we performed *in vitro* and *in vivo* studies. We found that midkine plays an important role in enhancement of HCC cell resistance to anoikis, thereby promoting subsequent metastasis. Activation of PI3K/Akt/NF-κB/TrkB signaling by midkine-activated anaplastic lymphomakinase (ALK) is responsible for anoikis resistance.

## INTRODUCTION

Hepatocellular carcinoma (HCC) is one of the most prevalent and lethal malignancies worldwide. Most HCC cases occur in the setting of a chronic liver disease, usually caused by infection with hepatitis B virus (HBV) or hepatitis C virus (HCV). The current modalities of curative treatment for HCC are limited to hepatectomy, local ablation, and liver transplantation. However, postoperative recurrence and metastasis are quite common, jeopardize overall survival, and finally lead to death in almost all patients [1]. Recurrence and metastasis arise through extremely complex molecular and pathological processes that generally require the dissemination of tumor cells from the primary lesion into the peripheral blood. The tumor cells disseminated into and circulating within the peripheral blood are called circulating tumor cells (CTCs) [2]. HCC shows a great tendency to spread locally and to invade blood vessels, particularly the portal vein, which underlies the clinical detection of CTCs in blood from the majority of patients with HCC [3, 4]. The fact that early tumor recurrence in liver transplanted HCC patients most commonly occurs in the newly implanted healthy liver allograft [5] strongly suggests a major contribution of CTCs to recurrence and metastasis after radical hepatectomy.

Anoikis is a critical mechanism for preventing ectopic cell growth or attachment to an inappropriate extracellular matrix, and also functions as a physiological barrier to cancer metastasis. Resistance to anoikis promotes tumor cell survival during the processes of local dissemination, systemic circulation and distant colonization [6]. It is conceivable that obtainment of anoikis resistance of CTCs is also a crucial event facilitating recurrence and metastasis of HCC. Several studies of HCC cell resistance to anoikis have investigated the potential involvement of multiple molecules and identified regulatory roles [7–14]. However, the mechanisms underlying resistance of HCC cells to anoikis are vastly unknown.

Midkine is a basic heparin-binding growth factor that is constitutively active when it forms homodimers that are stabilized by heparin. It is a highly conserved and developmentally regulated gene product, widely expressed in different cell types and strongly induced by retinoic acid during mid-gestation, hence the name “midkine” [15]. However, expression of midkine is extremely low and restricted to only a few cell types in adulthood [15, 16]. Different tyrosine kinase receptors, such as anaplastic lymphomakinase (ALK), have been identified as receptors of midkine [15, 17]. The interactions between midkine and these receptors have been shown to promote cell growth, survival, differentiation, migration, and angiogenesis [15]. It has been reported that midkine is overexpressed in at least 20 different types of cancers, ranging from the most common cancers to some of the rarest, and acts as a key factor associated with recurrent invasive and metastatic phenotypes of most malignant tumors [18, 19].

Several midkine studies have also provided evidence towards its functional role in HCC progression. In particular, midkine was shown to protect the HCC cell line (HepG2) against apoptosis [20]. Furthermore, the midkine gene was identified as one of the top five overexpressed genes in human HCC, with serum midkine levels being similarly significantly elevated [21]. A higher serum midkine level could be used for detecting early HCC and predicting metastasis and poor prognosis [19, 22, 23]. In addition, midkine-targeted technologies that inhibit midkine expression have been shown to effectively suppress HCC growth [24].

Based on these collected findings, we hypothesized that a higher blood midkine level might be required for resistance of HCC CTCs to anoikis, thereby facilitating survival in the systemic circulation and promoting recurrence and metastasis. Here, our present study demonstrates that midkine-induced anoikis resistance of HCC cells involves activation of its receptor ALK and an autocrine signaling loop, all of which are necessary for CTC survival and recurrence or metastasis formation.

## RESULTS

### Midkine confers anoikis resistance in HCC cells

The anoikis rates were lower for all of seven HCC cell lines examined than those for the two normal human liver cell lines examined at 24 hours post-midkine exposure (Supplementary Figure 1A, *p* < 0.05). Midkine mRNA expression level varied among the HCC cell lines tested, but the lowest expression level was detected in the normal human liver cell lines (Supplementary Figure 1B, *p* < 0.05). In addition, conditioned media from all the investigated HCC cell lines showed a significantly higher concentration of midkine protein (Supplementary Figure 1C, *p* < 0.05). As shown in Supplementary Figure 1A–1C, the differential expression profile of midkine for the various HCC cell lines followed a negative correlation to anoikis resistance. For example, the PLC/PRF/5 cells had the highest level of midkine protein and displayed the lowest anoikis rate. When incubated in suspension with midkine, HCC cells had significantly lower rates of anoikis; the results for Hep3B cells and PLC/PRF/5 cells are presented in Supplementary Figure 1D and Supplementary Figure 2A, respectively, showing that midkine significantly decreased anoikis in a dose-dependent manner (ranging from 10 to 80 ng/mL; *p* < 0.05).

### Involvement of midkine-mediated up-regulation of TrkB in anoikis resistance of HCC cells

**Figure 1:**
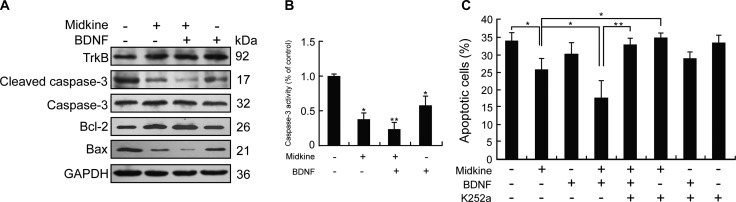
Tyrosine kinase receptor B (TrkB) is involved in midkine-mediated anoikis resistance of hepatocellular carcinoma (HCC) cells (**A**) Expression of TrkB and apoptosis-related proteins in Hep3B cells cultured with/without midkine (20 ng/mL) and brain-derived neurotrophic factor (BDNF, 10 ng/mL) for 24 hours, detected by Western blotting. (**B**) Caspase-3 activity was measured by a colorimetric assay based on the ability of caspase-3 to change Ac-DEVD-*p*NA into a yellow formazan product *p*NA. Activation level of caspase-3 was shown as a percentage of change in mean value derived from three separate experiments compared with control. (**C**) Anoikis rate of Hep3B cells treated with midkine (20 ng/mL) alone or TrkB ligand brain-derived neurotrophic factor (BDNF, 10 ng/mL) alone or a TrkB inhibitor (K252a, 300 nM) alone, or in combination. Data are presented as mean ± SD derived from three independent experiments. *p < 0.05, **p < 0.01.

In accordance with the observed midkine-induced anoikis resistance in Hep3B cells, the Bcl-2 representative anti-apoptotic expression profile was found to be up-regulated by midkine accompanied by down-regulated expressions of Bax and cleaved caspase-3 and up-regulated expression of TrkB, a potent anoikis suppressor (Figure 1A). Consistently, caspase-3 enzymatic activity was significantly decreased in midkine-treated cells (Figure 1B). PLC/PRF/5 cells also showed similar results (Supplementary Figure 2B and 2C). Furthermore, treatment of the Hep3B cells with BDNF (10 ng/mL, for 48 hours) alone, a known ligand of TrkB, up-regulated the expressions of TrkB and Bcl-2, down-regulated the expressions of Bax and cleaved caspase-3 as well as its activity (Figure 1A and 1B), and produced little effect on their anoikis rate (*p* > 0.05) (Figure 1C); when treatment with both midkine and BDNF, the rate of midkine-mediated anoikis was significantly decreased (*p* < 0.01) (Figure 1C) accompanied by up-regulated expression of TrkB and Bcl-2 (Figure 1A) but down-regulated expressions of Bax and cleaved caspase-3 as well as its activity (Figure 1A and 1B); in contrast, treatment with the TrkB inhibitor K252a (300 nM) increased both midkine-mediated anoikis rate and BDNF-mediated anoikis rate in the presence of midkine (*p* < 0.01) (Figure 1C).

### Autocrine function of ALK presence for midkine-mediated anoikis resistance, growth, and invasion in HCC cells

**Figure 2:**
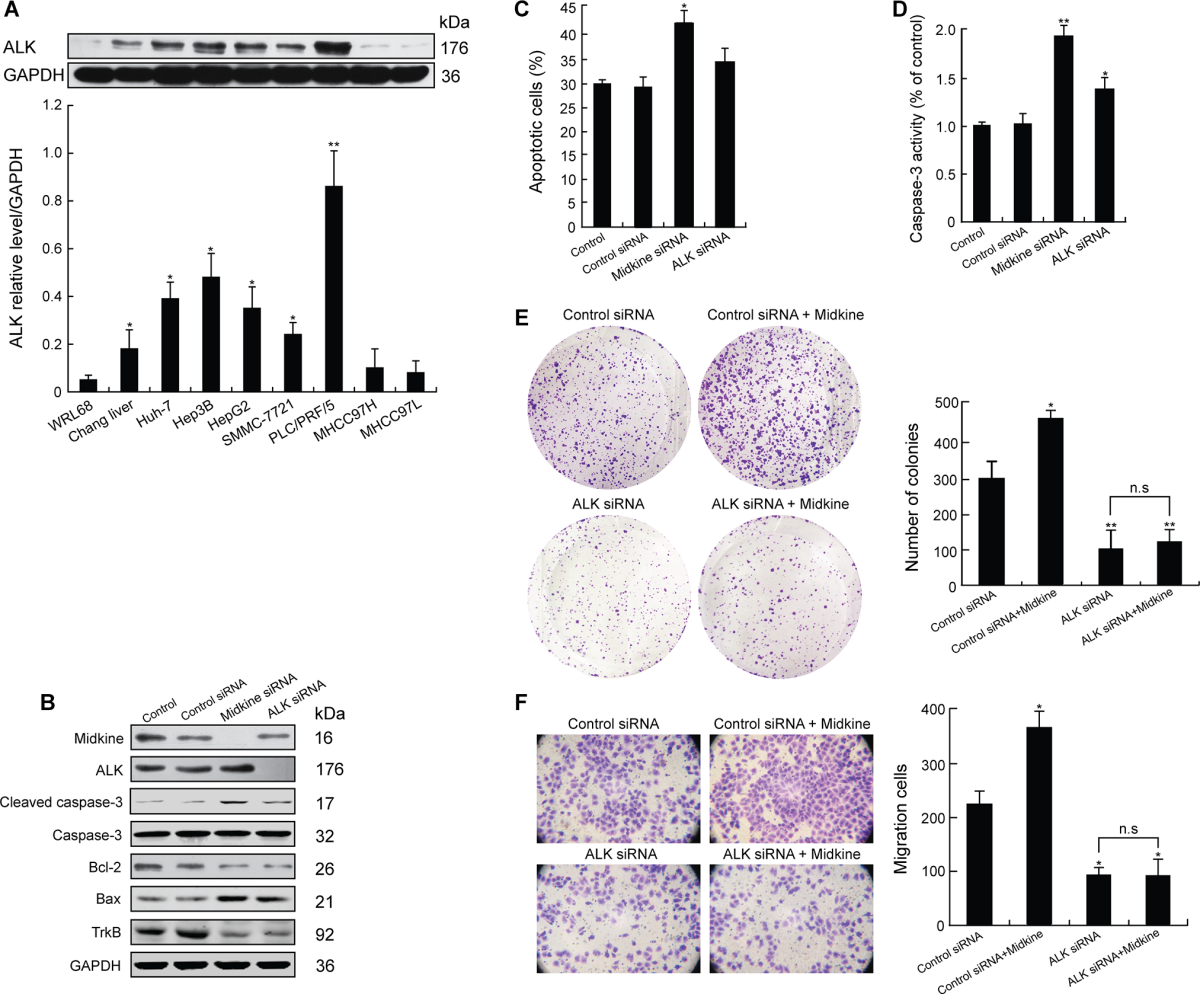
Midkine induces anoikis resistance, growth and invasion of hepatocellular carcinoma (HCC) cells through anaplastic lymphomakinase (ALK) activation (**A**) ALK expression in normal liver cell lines and various HCC cell lines detected by Western blotting. (**B**) Expression of tyrosine kinase receptor B (TrkB) and anti-apoptotic proteins in PLC/PRF/5 cells with knockdown of midkine or ALK expression assessed by Western blotting. (**C**) Anoikis rate of PLC/PRF/5 cells with knockdown of midkine or ALK expression. (**D**) Caspase-3 activity was determined with knockdown of midkine or ALK expression. Activation level of caspase-3 was shown as a percentage of change in mean value derived from three independent experiments compared with control. (**E**) Anchorage-independent growth of PLC/PRF/5 cells with knockdown of ALK expression assessed by soft agar colony formation assay. (**F**) Invasion ability of PLC/PRF/5 cells with knockdown of ALK expression assessed by transwell assay. Data are presented as mean ± SD derived from three independent experiments. *p < 0.05, **p < 0.01, n.s, not significant.

**Figure 3:**
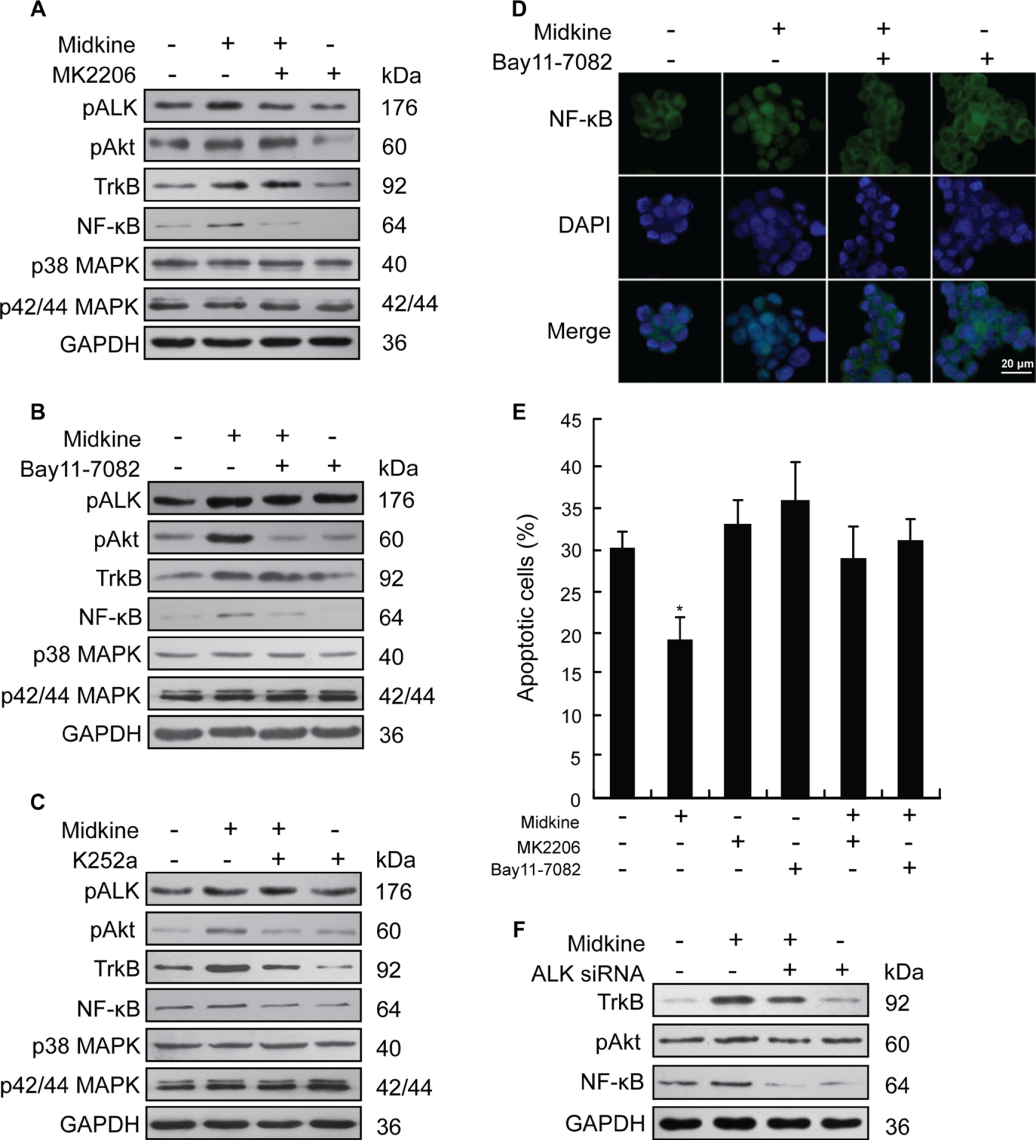
PI3K/Akt/NF-kB/TrkB signaling activated by anaplastic lymphomakinase (ALK) is required for midkine-induced anoikis resistance (**A**) Expression of signaling pathway-related proteins in suspension-cultured Hep3B cells treated with midkine (20 ng/mL) and protein kinase B (Akt) inhibitor MK2206 (10 μM), detected by Western blotting. (**B**) Expression of signaling pathway-related proteins in suspension-cultured Hep3B cells treated with midkine (20 ng/mL) and nuclear factor-kappaB (NF-kB) inhibitor Bay11-7082 (10 μM), detected by Western blotting. (**C**) Expression of signaling pathway-related proteins in suspension-cultured Hep3B cells treated with midkine (20 ng/mL) and tyrosine kinase receptor B (TrkB) inhibitor K252a (300 nM), detected by Western blotting. (**D**) Nuclear accumulation of NF-kB in suspension-cultured Hep3B cells treated with midkine (20 ng/mL) and Bay11-7082 (10 μM), assessed by spatial analysis. Blue: 4′,6-diamidino-2-phenylindole (DAPI); Green: NF-kB. Scale bar: 20 μm. (**E**) Anoikis rate of Hep3B cells treated with midkine (20 ng/mL), MK2206 (10 μM), and Bay11-7082 (10 μM). Data are presented as mean ± SD derived from three independent experiments. (**F**) Expression of TrkB, pAkt and NF-κB in Hep3B cells with midkine (20 ng/mL) alone or in combination with ALK siRNA assessed by Western blotting. *p < 0.05.

We next investigated the expression of ALK in the HCC cells. Western blot analysis indicated that 5 of the 7 HCC cell lines exhibited increased ALK expression (Figure 2A). As shown in Supplementary Figure 1B and 1C and in Figure 2A, PLC/PRF/5 cells expressed high levels of both midkine and ALK. We knocked down endogenous midkine or ALK expression in PLC/PRF/5 cells, and appropriate knockdown was confirmed by Western blotting (Figure 2B). In comparison with control siRNA-expressing cells, the cells with midkine knockdown or with ALK knockdown showed down-regulatied expressions of Bcl-2 and TrkB and up-regulated expressions of cleaved caspase-3 and Bax (Figure 2B), but the cells with midkine knockdown had significant sensitivity to anoikis (Figure 2C, *p* < 0.05). Accordingly, caspase-3 enzymatic activity was significantly increased in either midkine-knockdown cells (*p* < 0.01) or ALK-knockdown cells (*p* < 0.05) (Figure 2D). Unexpectedly, the cells with ALK knockdown showed a partial restoration of their sensitivity to anoikis (Figure 2C, *p* < 0.05), as well as a partial inhibition of anchorage-independent growth (Figure 2E, *p* < 0.01) in soft agar and invasion capacity across Matrigel^TM^ which occurred over a period of 16 hours of incubation in the poly-HEMA-coated transwell plates (Figure 2F, *p* < 0.05). Hep3B cells also showed similar results (Supplementary Figure 2D–2F). Interestingly, stimulation of suspension-cultured Hep3B cells with midkine for 48 hours induced an increase in pALK expression (Figure 3A). Furthermore, cells with ALK knockdown showed a poor response to exogenously added midkine for promotion of anchorage-independent growth, invasion, and anoikis as compared with the cells expressing control siRNA (Figure 2E and 2F).

### Effect of midkine-activated ALK on PI3K/Akt/NF-κB/TrkB signaling is required for midkine-induced anoikis resistance

Next, Hep3B cells were used to identify the signaling pathway that mediates midkine-induced anoikis resistance, which expressed a lower level of midkine and a higher level of ALK as shown in Supplementary Figure 1B, 1C, and Supplementary Figure 2A. Stimulation of suspension-cultured Hep3B cells with midkine for 48 hours did not cause a significant increase in p42/44 MAPK and p38 MAPK but induced an increase in the expression of pALK, pAkt and NF-κB (Figure 3A–3C), which was accompanied by a nuclear accumulation of NF-κB (Figure 3D). In cells with ALK knockdown, by contrast, exposure to midkine did not induce a significant increase in expression of either pAkt or NF-κB (Figure 3F). Pretreatment with the Akt inhibitor MK2206 (10 μM) for 48 hours led to a greater amount of the midkine-treated Hep3B cells being sensitive to anoikis (Figure 3E, *p* < 0.05), and this effect was accompanied by decreased expressions of pAkt, NF-κB and TrkB (Figure 3A–3C). Treatment with the NF-κB inhibitor Bay11-7082 (10 μM) for 48 hours also led to a greater amount of midkine-treated Hep3B cells being susceptible to anoikis (Figure 3E, *p* < 0.05), which was accompanied by partial decrease in expression of NF-κB and TrkB and significant decrease in pAkt expression (Figure 3A–3C). Treatment of Hep3B cells with the TrkB inhibitor K252a led to a similar slight decrease in the expression of pAkt and NF-κB (Figure 3A–3C). Similar results were observed in parallel assays performed with PLC/PRF/5 cells (Supplementary Figure 3). Experiments of Hep3B cells with siRNA targeting Akt, NF-κB and TrkB also revealed changes similar to those observed in the presence of the inhibitors of MK2206, Bay11-7082 and K252a (Supplementary Figure 4).

### Midkine promotes CTC survival and tumor metastasis in mice

**Figure 4:**
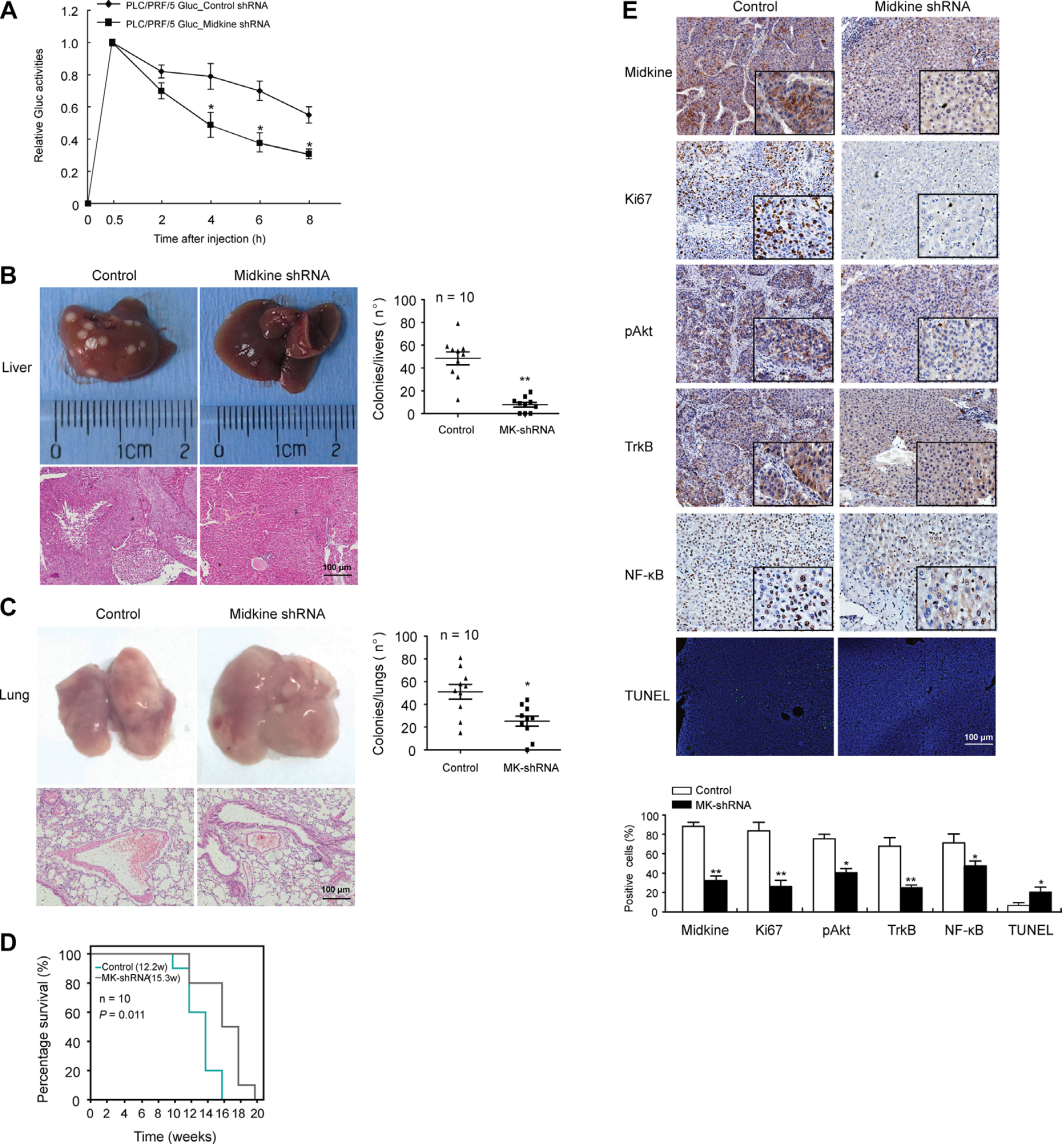
Midkine promotes circulating tumor cell (CTC) survival and tumor metastasis in mice (**A**) PLC/PRF/5 cells and control cells with Gluc stably-silenced midkine were injected into mice (*n* = 10) through tail vein. Gluc activity was determined to assess anoikis of injected cells *in vivo*. (**B**) The mice injected with midkine-knockdown cells (*n* = 10) had a significantly smaller number of tumor foci on the surface of liver compared to control mice (*n* = 10). (**C**) The mice injected with midkine-knockdown cells (*n* = 10) had a significantly smaller number of tumor foci on the surface of lung compared to control mice (*n* = 10). (**D**) The mice injected with midkine-knockdown cells (*n* = 10) had a longer survival time compared to control mice (*n* = 10). (**E**) Representative expressions of midkine, Ki67, phosphorylated protein kinase B (pAkt), tyrosine kinase receptor B (TrkB), and nuclear factor-kappaB (NF-kB) detected by immunohistochemical staining, and representative cell apoptosis detected by TUNEL assay in liver tumors from different mice. Scale bar: 100 μm. Data are presented as mean ± SD derived from three independent experiments. *p < 0.05, **p < 0.01.

*In vivo* studies were conducted comparing stable midkine knockdown PLC/PRF/5 cells with control shRNA-expressing cells that had been introduced into nude mice via tail vein injection. Mice with the shRNA-silenced endogenous midkine showed a dramatically lower level of Lv-Gluc in peripheral blood (Figure 4A, *p* < 0.05), suggesting that midkine functions as a promoter of the anoikis resistant phenotype of HCC cells *in vivo* to protect from CTC death during circulation. Furthermore, at 3 weeks after the tail vein injection conducted with a hydrodynamics-based procedure [25, 26], compared to the control mice, the mice injected with midkine-knockdown cells had a significantly greater number of tumor foci on the surface of liver (*p* < 0.01) plus lung (*p* < 0.05) (Figure 4B and 4C), and a longer survival time (Figure 4D, *p* = 0.011). These data indicated that midkine-induced protection from cell death increased homing and colonization of HCC cells in liver and lung, and promoted their growth and metastasis formation in secondary sites.

Tumor occurrence in liver and lung was evidenced by H&E staining (Figure 4B and 4C). Immunohistochemical and TUNEL staining showed that lower expression of midkine protein was accompanied by lower expression of Ki67 (a nuclear marker for proliferation) in liver tumors from mice injected with midkine-knockdown cells (Figure 4E, *p* < 0.01); in addition, these tumors showed a higher extent of TUNEL staining (*p* < 0.05) and a decreased expression level of pAkt (*p* < 0.05), TrkB (*p* < 0.01) and NF-κB (*p* < 0.05) (Figure 4E).

### Serum midkine level was significantly associated with both CTC counts and postoperative recurrence in HCC patients

The median serum level of midkine in the 341 HCC patients was 0.862 ng/mL (range, 0.215–1.583 ng/mL), significantly higher than that in the 78 healthy individuals (0.138 ng/mL; range, 0.106–0.317 ng/mL, *p* < 0.001); additionally, 238 of the patients with HCC (69.8%) had an elevated serum level of midkine (≥ 0.5 ng/mL). CTCs and apoptotic CTCs were simultaneously detected in all patients with HCC, and data from a representative patient are shown in Supplementary Figure 5. CTCs were detected in 276 of the 341 (80.9%) total patients with HCC, with a median CTC count of 16.5 ± 9.7 CTCs/5 mL. We chose 102 HCC samples randomly from among the total 341 samples for apoptotic CTC evaluation. The blood samples of midkine-elevated subtypes had significantly higher CTC counts than those of midkine-normal subtypes (19.5 ± 10.4 CTCs *vs*. 14.8 ± 7.1 CTCs, *p* = 0.012). More apoptotic CTC counts were found in patients with midkine-normal levels compared with those in the group of patients with midkine-elevated levels (10.1 ± 3.7 CTCs *vs*. 6.2 ± 2.7 CTCs, *p* = 0.000). The median percentage of apoptotic CTCs in the total analyzed CTCs per patient was 31.2% (range, 0–100%) in patients with midkine-elevated levels and 69.6% (range, 0–100%) in patients with midkine-normal levels (Supplementary Table 2). Furthermore, patients with midkine-elevated levels had a significantly higher recurrence rate compared to those with normal levels, and the recurrence interval of the midkine-elevated subset was significantly shorter (18.6 ± 5.2 months *vs*. 21.6 ± 4.8 months, *p* = 0.001) (Table 1).

**Table 1:** Comparison of recurrence in patients with HCC and midkine-elevated or midkine-normal levels

	Midkine-elevated (*n* = 238)	Midkine-normal (*n* = 103)	*p*
Recurrence, *n* (%)	182 (76.5)	63 (61.2)	0.004
Intrahepatic recurrence, *n* (%)	147 (61.8)	48 (46.6)	0.009
Extrahepatic recurrence, *n* (%)	58 (24.4)	12 (11.7)	0.008
Intra+extrahepatic recurrence, *n* (%)	60 (25.2)	17 (16.5)	0.078
Interval of recurrence, months	18.6 ± 5.2	21.6 ± 4.8	0.001

## DISCUSSION

Resistance to anoikis is known as an important step in metastasis, as it affords tumor cells increased survival times while migrating to secondary sites [6]. Conceivably, CTCs are challenged by anoikis during their systemic circulation. Several circulating cytokines, including hepatocyte growth factor (HGF) [27], TGF-beta [28], CXCL8 [29] and CXCL12 [30], have been implicated as contributors to anoikis signaling in some kinds of tumor cells. However, little is known about the role of cytokines in anoikis signaling in HCC cells. In the current study, we addressed how and whether midkine is directly involved in resistance of CTCs to anoikis, and found that midkine plays an important role in enhancement of HCC cell resistance to anoikis, which was further demonstrated by an increased level of Bcl-2 and TrkB expressions and a decreased level of Bax and cleaved caspase-3 expressions as well as caspase-3 enzymatic activity.

ALK gene copy number is frequently detected in clinical evaluation of solid tumors such as non-small cell lung cancer, neuroblastoma, esophageal cancer and HCC, and has been associated with a subgroup of high-risk neuroblastoma and HCC [31, 32]. Furthermore, ALK is a receptor for midkine, through which midkine contributes to glioma progression and thus renders glioma cells resistant to autophagy-mediated cell death and antitumoral effects of cannabinoids [33, 34]. We investigated whether ALK is involved in midkine-mediated anoikis resistance of HCC cells, the combined data of the experiments with both exogenous midkine induction and ALK knockdown suggest a possible autocrine function of ALK presence for midkine-mediated growth, invasion, and anoikis resistance in HCC cells. Recent studies have examined ALK expression in HCC tissues by IHC and report 44.7% (153/342) [35] and 13.15% (28/213) [32] for ALK expression, which is much lower than that (5/7, 71.4%, Figure 2A) of the HCC cell lines examined in the present study. It should be noted that the concomitant expressions of midkine and ALK are not found in all the examined HCC cell lines, for example, PLC/PRF/5 cells show the maximum expression in both midkine and ALK, whereas MHCC97 cells, known as the most metastatic cells, expresses a higher level of midkine but a very low level of ALK. The heterogeneous expression profiles of midkine and ALK may imply that midkine-induced anoikis resistance in some HCC cells is independent of ALK activation, and probably involved in an alternative mechanism of activating another unique pathway.

In the current study, the PI3K/Akt pathway was required for midkine-induced anoikis resistance that involved midkine-mediated ALK signaling in HCC cells, as evidenced by the results that suspension-cultured HCC cells treated with exogenous midkine showed an increase in pAkt expression; pretreatment with the Akt inhibitor MK2206 or Akt-specific siRNA effectively inhibited midkine-mediated anoikis resistance, and this response was accompanied by decreased expression of pAkt; consistently, in cells with ALK knockdown, midkine did not induce a significant increase in pAkt expression.

TrkB, a potent anoikis suppressor, is overexpressed in several kinds of malignancies, and BDNF-mediated activation of TrkB (ligand binding to the receptor) contributes to anoikis resistance of cancer cells, including HCC [36]. In the current study, we found that midkine-mediated anoikis resistance of HCC cells was accompanied by up-regulated expression of TrkB, and that midkine knockdown resulted in down-regulation of TrkB. Moreover, BDNF enhanced midkine-mediated anoikis resistance, while treatment with the TrkB inhibitor K252a or TrkB-specific siRNA made HCC cells sensitive to anoikis mediated by both midkine and BDNF in the presence of midkine and also led to a slight decrease in the expressions of pAkt. These results indicate that midkine up-regulates TrkB expression, and activated TrkB in turn up-regulates pAkt expression, which contributes to midkine-mediated anoikis resistance of HCC cells.

NF-κB is best known as a transcription factor, and has been shown to play a role in apoptosis by regulating genes involved in cell death [37]. In the current study, treatment with either the Akt inhibitor MK2206 or Akt-specific siRNA led to down-regulation of NF-κB expression in HCC cells; intriguingly, treatment with either the NF-κB inhibitor Bay11-7082 or NF-κB siRNA also led to down-regulation of the expressions of TrkB and pAkt, and this process was accompanied by abrogation of midkine-induced anoikis resistance. These results suggest that an autocrine signaling loop of PI3K/Akt/NF-κB/TrkB/PI3K/Akt is involved in midkine-induced anoikis resistance, and NF-κB is a major signal transducer in this signal loop.

**Figure 5:**
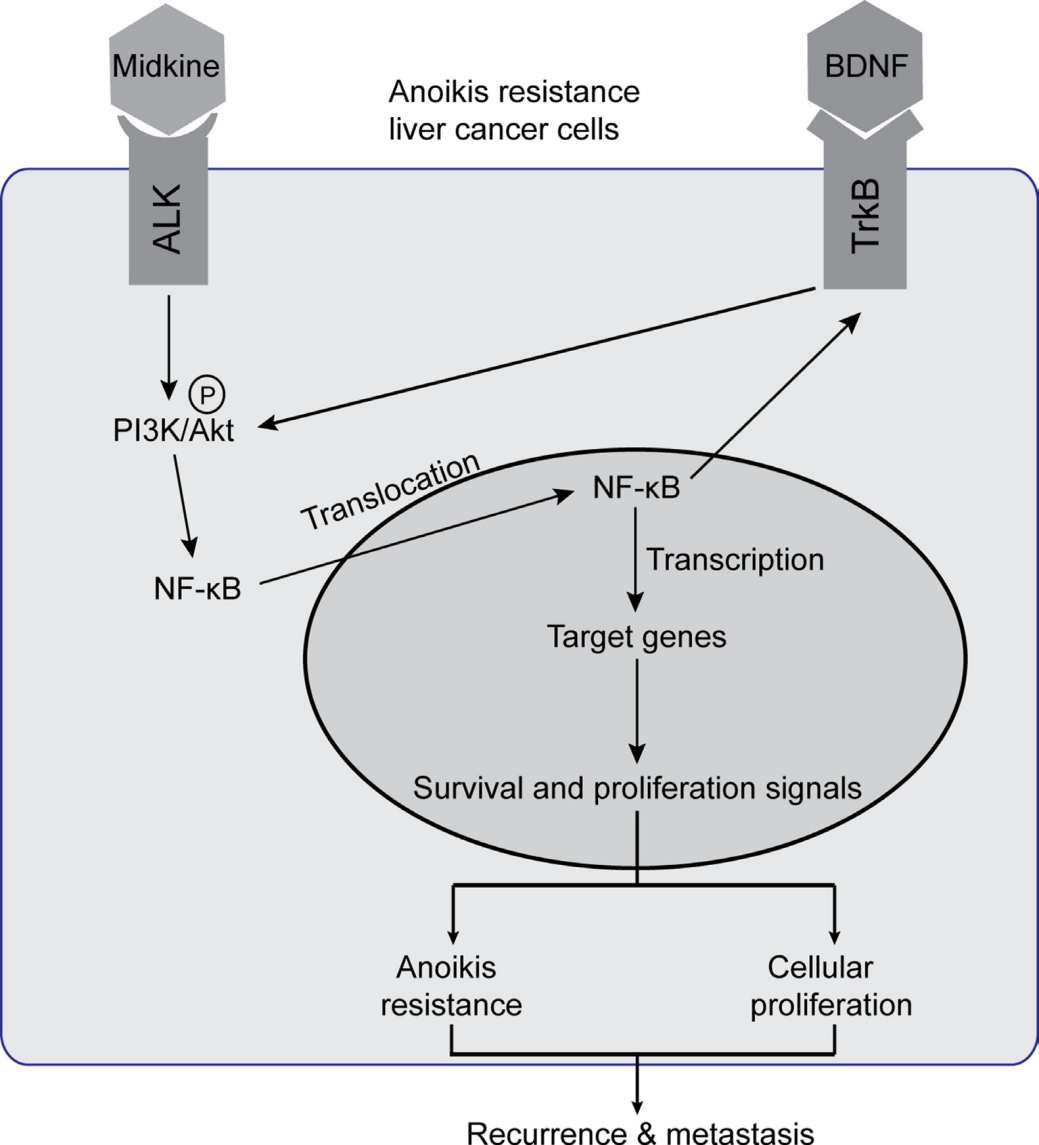
Schematic illustration of signaling pathways activated in midkine-mediated anoikis resistant hepatocellular carcinoma (HCC) cells Endogenous or exogenous midkine activates anaplastic lymphomakinase (ALK, receptor of midkine), which in turn promotes phosphorylation of protein kinase B (pAkt). When pAkt is expressed at high levels, NF-kB in the cytoplasm enters into the nucleus to regulate transcription of its target genes and consequently induce HCC cells anoikis resistance and proliferation. Meanwhile, nuclear factor-kappaB (NF-kB) up-regulates tyrosine kinase receptor B (TrkB), the neurotrophic tyrosine kinase receptor 2 which acts as a potent anoikis suppressor and may enhance phosphorylation of Akt to facilitate a positive feedback loop. Brain derived neurotrophic factor (BDNF), a known ligand of TrkB, promotes midkine-mediated anoikis resistance with similar effects.

Collectively, the findings from the present study provide an overview, as depicted in Figure 5, of midkine-stimulated and suspended HCC cells in which the midkine-ALK reaction activates the PI3K/Akt pathway leading to activation of NF-κB signaling. The subsequent increase in NF-κB transcriptional activity mediates up-regulation of TrkB, which itself is activated by its ligand BDNF signal through pAkt to enhance NF-κB activation, establishing an autocrine signaling loop. In this signal loop, midkine is a mediator of ALK activation, and NF-κB is a major signal transducer that induces transcription of genes to facilitate anoikis resistance or proliferation, ultimately leading to protection from CTC death during circulation and promotion of subsequent tumor recurrence and metastasis, as demonstrated by the results from our animal studies and correlative studies in HCC patients. Finally, our characterization of an autocrine signaling loop in midkine-induced anoikis resistant HCC cells develops a framework for advancing combinatorial therapeutic strategies. Inhibition of midkine along with an NF-κB inhibitor targeting HCC cells (such as CTCs in different stages over the course of disease progression) may represent a novel combinatorial strategy to improve the therapeutic outcome of HCC.

## MATERIALS AND METHODS

### Cell culture and treatment

The liver cancer cell lines PLC/PRF/5, Huh 7, Hep3B, HepG2, SMMC7721, MHCC97L and MHCC97H were obtained and cultured as previously described [3, 38]. For pharmacological inhibition, cells cultured with/without 10 μM of midkine (R&D Systems, Minneapolis, MN, USA) were treated with either 10 μM of the Akt inhibitor MK2206 (Selleck Chemicals, Houston, TX, USA), 300 nM of the tyrosine kinase receptor B (TrkB) inhibitor K252a (Calbiochem, Darmstadt, Germany), or 10 μM of the NF-κB inhibitor Bay11-7082 (Sigma-Aldrich, St. Louis, MO, USA) for the indicated durations of time. Each of the compounds (K252a, Bay11-7082, and MK2206) had been dissolved in 100% dimethyl sulfoxide (DMSO) (Sigma-Aldrich), with the final concentration of 0.1% diluted with Dulbecco's modified Eagle's medium (DMEM) (Gibco of Thermo Fisher Scientific, Waltham, MA, USA). For stimulation with brain-derived neurotrophic factor (BDNF), cells cultured with/without midkine were treated with/without 10 ng/mL recombinant human BDNF (Peprotech, Rocky Hill, NJ, USA) for 48 hours.

### Quantitative reverse transcription-PCR (qRT-PCR)

The following primers were used for the analysis of midkine: forward primer, 5′-ATGC AGCACCGAGGCTTCCT-3′; reverse primer, 5′-TTCCCT TCCCTTTCTTGGCTT-3′. Glyceraldehyde-3-phosphate dehydrogenase (GAPDH) was amplified as an endogenous control using the following primers: forward primer, 5′-TCACCAGGGCTGCTTTTAAC-3′; reverse primer, 5′-GACAAGCTTCCCGTTCTCAG-3′. The REST 2009 V2.0.13 software (Qiagen, Hilden, Germany) was used for calculation and analysis of the detected midkine gene expression.

### Anoikis, colony formation and matrigel invasion assays

For induction of anoikis, 6-well tissue culture plates were treated with poly-hydroxyethyl methacrylate (HEMA) (Sigma-Aldrich) and used with cells cultured under suspension conditions. After harvesting, cell pellets were fixed and stained with Annexin V/propidium iodide using the TACSÒ Apoptosis Detection Kit (Trevigen, Inc., Gaithersburg, MD, USA), and the presence of apoptotic cells was analyzed within 1 hour by flow cytometry using an Epics Profile flow cytometer (Coulter, Miami, FL, USA). Soft agar assay and invasion assay were performed according to the methods described previously [39].

### Western blot analysis

Primary antibodies were as follows: mouse anti-human TrkB, mouse anti-human ALK, mouse anti-human caspase-3 and cleaved caspase-3, mouse anti-human Bcl-2, mouse anti-human Bax, mouse anti-human NF-κB (p65) (Abcam, Cambridge, UK), rabbit anti-human pAkt (Ser473), rabbit anti-human pALK (Tyr1507), rabbit anti-human Akt (C67E7), rabbit anti-human p38 MAPK (D13E1), rabbit anti-human pERK1/2 (Thr202/Tyr204), and rabbit anti-human ERK1/2 (137F5) (Cell Signaling Technology, Danvers, MA, USA). GAPDH (detected as a loading control), mouse anti-human Midkine and all secondary antibodies were purchased from Santa Cruz Biotechnology (Dallas, TX, USA). The primary and secondary antibodies were used at 1/2000 dilution. Immunoreactive proteins were visualized using an enhanced chemiluminescence detection system (Sigma-Aldrich) with exposure to X-ray film. The resultant film images were analyzed by Quantity One analysis software (Bio-Rad, Hercules, CA, USA). The results were quantified by normalization of three separate experiments to GAPDH values.

### Caspase-3 activity assay

Caspase-3 enzymatic activity in cell lysates was measured using a Caspase-3 Activity Detection kit (Beyotime, Jiangsu, China) according to the manufacturer's protocol.

### Silencing of midkine and ALK with siRNA

Midkine, ALK, Akt, NF-κB, and TrkB were knocked-down by transfecting SMARTpool siRNAs targeting each respectively (GE Healthcare, Pittsburgh, PA, USA) into cell lines using the LipofectAMINE 2000 reagent (Life Technologies, Carlsbad, CA, USA) according to the manufacturer's protocol.

### Spatial analysis of NF-κB

NF-κB was detected by using the NF-κB Activation Nuclear Translocation Assay Kit (Beyotime, Jiangsu, China), and the nucleus was identified by 4′,6-diamidino-2-phenylindole (DAPI) staining (Beyotime).

### HCC CTC enrichment, enumeration and characterization

CTC enrichment and detection in whole blood samples of HCC patients were conducted according to the method described previously [3]. Cells that were CD45-negative, asialoglycoprotein receptor (ASGPR)-positive or/and carbamoyl phosphate synthetase 1 (CPS1)-positive and DAPI-stained that met morphologic features of malignant cells (large cellular size, high nucleus to cytoplasm ratio, and visible nucleoli) were scored as HCC CTCs. CTC counts are presented herein as the number of cells per 5 mL of blood.

M30 is a caspase-cleaved fragment of cytokeratin 18 (CK18), and is routinely used in laboratory and clinic as a biomarker of tumor cell apoptosis [40]. To specifically quantify apoptotic CTCs, M30-positive CTCs were detected by the M30 CytoDEATH^TM^ Fluorescein Assay Kit (Peviva, Stockholm, Sweden), which recognizes a neoepitope disclosed by caspase cleavage at CK18 during early apoptosis [40]. The result is expressed herein as the total number of CTCs and M30-positive CTCs per 5 mL of blood.

### ELISA

The assay was run using the Human Midkine DuoSet ELISA Development Kit (R&D Systems) according to the manufacturer's protocol.

### Tumor xenograft experiment

The animal welfare guidelines for the care and use of laboratory animals were followed and the experimental protocol was approved by the Animal Care Committee of Second Military Medical University (Shanghai, China). PLC/PRF/5 cells, with/without midkine knockdown (Midkine shRNA, Santa Cruz Biotechnology, Dallas, TX, USA), were used to generate liver tumors in BALB/c nude mice (Shanghai Laboratory Animal Center, Chinese Academy of Sciences, Shanghai, China). All mice were cared for in accordance with the appropriate institutional guidelines. Anoikis *in vivo* was assessed by quantitative assay of cellular Gluc activity in blood [7]. Briefly, PLC/PRF/5 cells (1 × 10^6^) infected with Lv-Gluc-shMidkine (Gauss Luciferase; GeneCopoeia, Rockville, MD, USA) were injected through the tail vein of the BALB/c mice. Thirty minutes after the injection, blood was collected every 2 hours using the method for sequential blood sampling by tail incision with photo monitoring, a method used for measurement of CTC clearance rate from bloodstream [7, 41]. The collected 5 μL of blood was added to 1 μL of 20 mM ethylene diamine tetraacetic acid (EDTA) and Gluc activity was measured using a TD 20/20 luminometer (Turner Design Inc., Sunnyvale, CA, USA) which was set to inject 100 μL of 100 μM coelenterazine (Goldbio, St. Louis, MO, USA) in DMEM and to acquire photon counts for 10 seconds.

To prepare a liver-specific tumor model induced by CTCs, a large volume of tumor cell solution was injected into the mouse tail vein using a hydrodynamics-based procedure [25, 26]. Three weeks after the injection, tumor-bearing mice were sacrificed by cervical dislocation and blood samples were collected from the inferior vena cava, in addition the main organs were removed, photographed and fixed. Whole blood was processed within 1 hour of collection. CTCs were isolated as described previously [42] and detected by a previously developed method [43].

### Histological analysis

The TUNEL assay for apoptosis detection was performed with the DeadEnd^TM^ Fluorometric TUNEL System (Promega, Madison, WI, USA). For immunohistochemistry (IHC), the tissue sections were consecutively incubated with the unconjugated primary antibodies for midkine, Ki67, pAkt, TrkB and NF-κB and the appropriate biotinylated secondary antibodies (Maixin-Bio, Fuzhou, China).

### Patients and specimens

Patients with HCC who underwent curative partial hepatectomy between October 2010 and March 2012 were recruited to the study. Clinical characteristics of the 341 patients who enrolled in the study are summarized in Supplementary Table 1. Blood was drawn by venipuncture into serum-separator tubes for midkine measurement and into VACUETTE^®^ polyethylene tubes containing EDTA (Greiner Bio-One GmbH, Frickenhausen, Germany) for CTC detection. Long-term follow-up of these patients was carried out as described previously [38]; the median follow-up time was 20.0 months (range, 1.5–36.0 months). The use of human tissue samples and clinical data was approved by the Biomedical Ethics Committee of Eastern Hepatobiliary Surgery Hospital, Second Military Medical University (Shanghai, China). All patients provided the informed written consent.

### Statistical analyses

The statistical analyses were performed using the SPSS Statistical Software Package V17.0 (SPSS, Inc., Chicago, IL, USA). A two-sided *p*-value of 0.05 or less was considered statistically significant. Comparison of categorical variables was performed using the chi-square test. Spearman's rank correlation analysis was used to determine nonparametric correlation. Rate of progression-free survival was estimated using the Kaplan-Meier method, and comparison of survival rates among groups was conducted using the log-rank test.

## SUPPLEMENTARY MATERIALS FIGURES AND TABLES



## Abbreviations

ALK: anaplastic lymphomakinase
ASGPR: asialoglycoprotein receptor
BDNF: brain derived neurotrophic factor
CPS1: carbamoyl phosphate synthetase 1
CTCs: circulating tumor cells
DAPI: 4′,6-diamidino-2-phenylindole
GAPDH: glyceraldehyde 3-phosphate dehydrogenase
HCC: hepatocellular carcinoma
HEMA: hydroxyethyl methacrylate
NF-κB: nuclear factor-kappaB
qRT-PCR: quantitative reverse transcriptase polymerase chain reaction
siRNA: small interfering RNA
TrkB: tyrosine kinase receptor B
