# Supplementary material for: Midkine promotes hepatocellular carcinoma metastasis by elevating anoikis resistance of circulating tumor cells
Source: Oncotarget. 2017 Mar 1;8(20):32523–35. doi: 10.18632/oncotarget.15808 (PMC5464806; doi:10.18632/oncotarget.15808)
Supplement: Supplementary file 1 [file oncotarget-08-32523-s001.pdf]

Midkine promotes hepatocellular carcinoma metastasis by elevating anoikis resistance of circulating tumor cells

Supplementary Materials

Experimental Procedures

Soft agar assay

For soft agar assay, trypsinized cells were seeded in 0.4% low melting-point agarose (Sigma-Aldrich, St. Louis, MO, USA) on top of a 1% agarose layer, and scans were made 11 days later. The numbers of macroscopic colonies were determined using Image J software (http://rsb.info.nih.gov/ij/index.html).

Invasion assay

For invasion assay, cells were cultured with midkine in transwell chambers (Corning Inc., Corning, NY, USA) coated with Matrigel™ (BD Diagnostics, Franklin Lakes, NJ, USA). Quantification was performed by counting the number of invaded cells on five independent pictures of the well.

HCC CTC enrichment, enumeration, and characterization

Following density gradient centrifugation with Ficoll-Paque PLUS (GE Healthcare Life Sciences, Little Chalfont, Buckinghamshire, United Kingdom), CTCs were enriched by extracting CD45-expressing leukocytes with magnetically labeled anti-CD45 monoclonal antibody (Miltenyi Biotec GmbH, Bergisch Gladbach, Germany) according to the instructions. The remaining cells were cytocentrifuged on polylysine-coated slides. Slides were coincubated with a mouse monoclonal antibody cocktail against asialoglycoprotein receptor (ASGPR) and carbamoyl phosphate synthetase 1 (CPS1) (Abcam, MA, USA) and a rat anti-human CD45 monoclonal antibody (Santa Cruz Biotechnology, Dallas, TX, USA), followed by incubation with a Cy3-conjugated goat anti-mouse IgG antibody and an Alexa Fluor 488-conjugated rabbit anti-rat IgG antibody (Invitrogen, Carlsbad, CA, USA), and subsequent costaining with DAPI. Stained slides were assessed by fluorescence microscopy (IX71; Olympus, Tokyo, Japan), and images were captured from positive-stained and control specimens using the same detector sensitivity and exposure time.

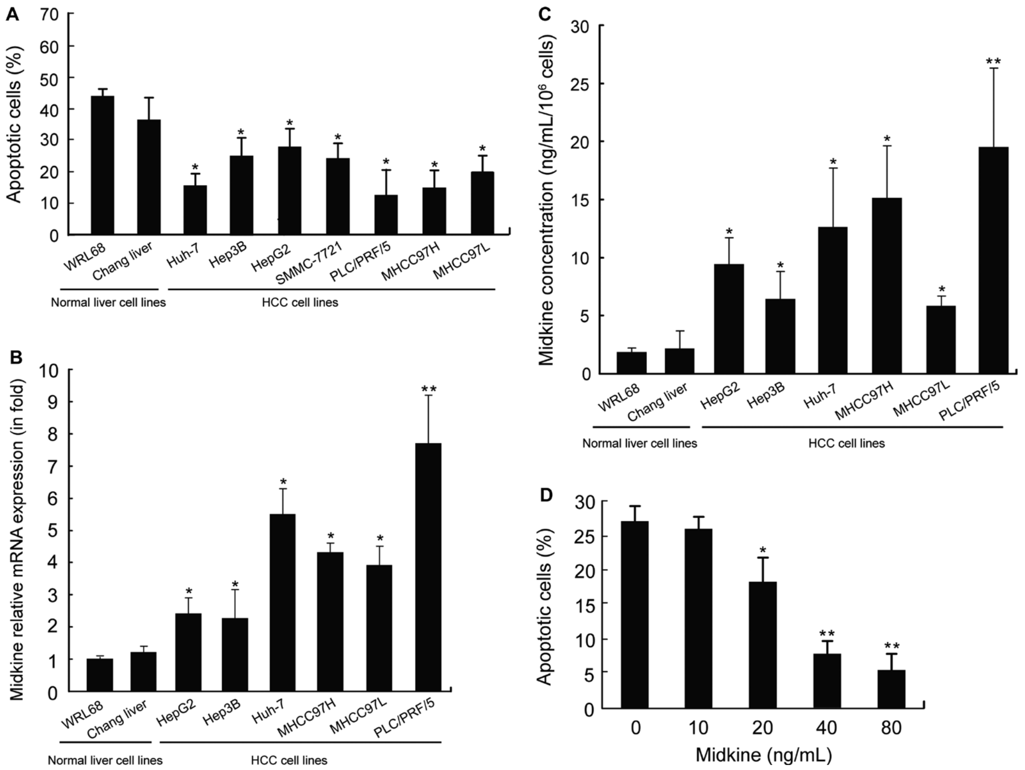

Supplementary Figure 1: Midkine confers anoikis resistance in hepatocellular carcinoma (HCC) cells. (**A**) The anoikis rates of 7 HCC cell lines and 2 normal human liver cell lines examined at 24 hours post-midkine exposure (20 ng/mL). All 7 HCC cell lines showed lower 24-h anoikis rates than WRL68 and Chang liver. (**B**) All 7 HCC cell lines showed higher relative expression levels of midkine mRNA than the normal human liver cell lines, measured by qRT-PCR. (**C**) Conditioned media from all HCC cell lines showed significant higher concentrations of midkine protein than the normal human liver cell lines, determined by ELISA. (**D**) Anoikis rate of Hep3B cells in response to treatment with different concentrations of midkine. Data are presented as mean ± SD derived from three independent experiments. *$p < 0.05$, **$p < 0.01$.

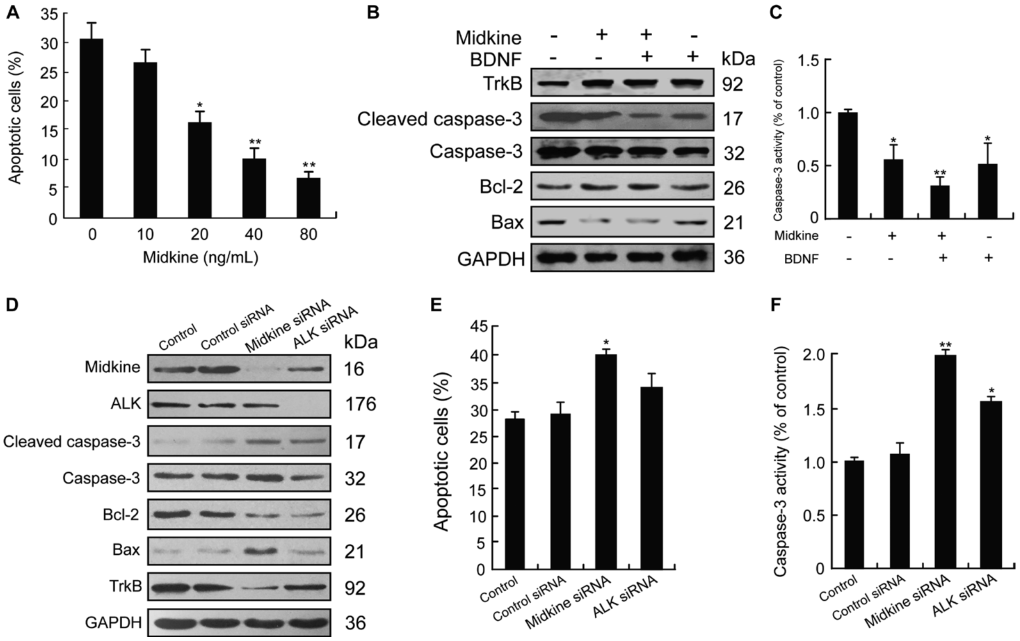

Supplementary Figure 2: Midkine induces anoikis resistance in hepatocellular carcinoma (HCC) cells. (**A**) Anoikis rate of PLC/PRF/5 cells in response to treatment with different concentrations of midkine. (**B**) Expression of TrkB and apoptosis-related proteins in PLC/PRF/5 cells cultured with/without midkine (20 ng/mL) and/or brain-derived neurotrophic factor (BDNF, 10 ng/mL) for 24 hours, detected by Western blotting. (**C**) Caspase-3 activity was measured in PLC/PRF/5 cells cultured with/without midkine (20 ng/mL) and/or brain-derived neurotrophic factor (BDNF, 10 ng/mL) for 24 hours. (**D**) Expressions of tyrosine kinase receptor B (TrkB) and anti-apoptotic proteins in Hep3B cells with midkine knockdown or ALK knockdown assessed by Western blotting. (**E**) Anoikis rate of Hep3B cells with midkine knockdown or ALK knockdown. (**F**) Caspase-3 activity was measured in Hep3B cells with midkine knockdown or ALK knockdown. Data are presented as mean ± SD derived from three independent experiments. *$p < 0.05$, **$p < 0.01$.

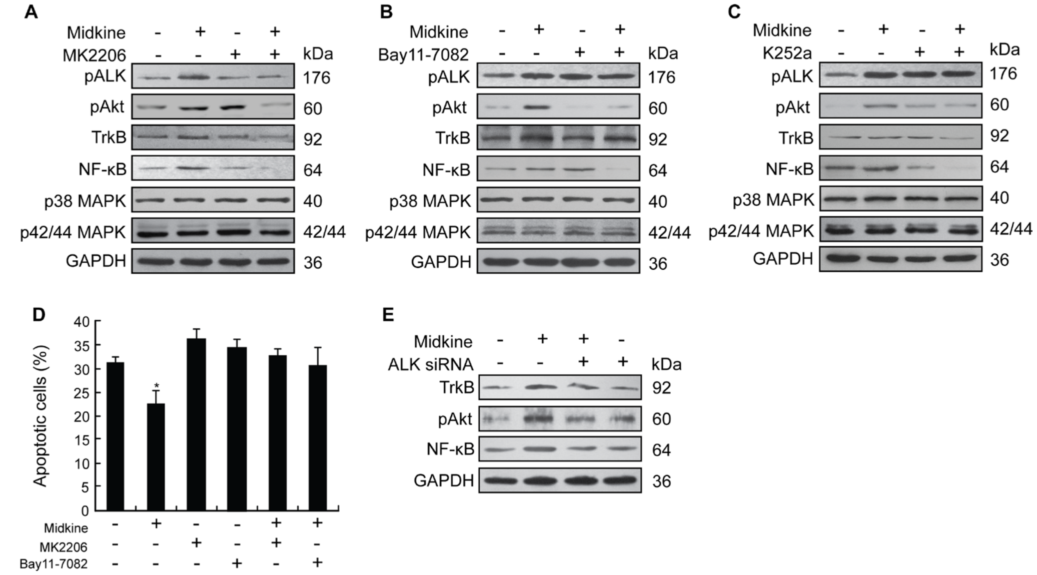

Supplementary Figure 3: PI3K/Akt/NF-κB/TrkB signaling activated by anaplastic lymphomakinase (ALK) is required for midkine-induced anoikis resistance of PLC/PRF/5 cells. (**A**) Expressions of signaling pathway-related proteins in suspension-cultured PLC/PRF/5 cells treated with/without midkine (20 ng/mL) alone or with protein kinase B (Akt) inhibitor MK2206 (10 μM) alone, or in combination for 24 hours, detected by Western blotting. (**B**) Expressions of signaling pathway-related proteins in suspension-cultured PLC/PRF/5 cells treated with/without midkine (20 ng/mL) alone or with NF-κB inhibitor Bay11-7082 (10 μM) alone, or in combination for 24 hours, detected by Western blotting. (**C**) Expressions of signaling pathway-related proteins in suspension-cultured PLC/PRF/5 cells treated with/without midkine (20 ng/mL) alone or with TrkB inhibitor K252a (300 nM) alone, or in combination for 24 hours, detected by Western blotting. (**D**) Anoikis rate of PLC/PRF/5 cells treated with/without midkine (20 ng/mL) alone, MK2206 (10 μM) alone, or Bay11-7082 (10 μM) alone, or in combination for 24 hours. (**E**) Expressions of TrkB, pAkt, and NF-κB in PLC/PRF/5 cells treated with/without midkine (20 ng/mL) alone or in combination with ALK siRNA for 24 hours, assessed by Western blotting. Data are presented as mean ± SD derived from three independent experiments. *$p < 0.05$.

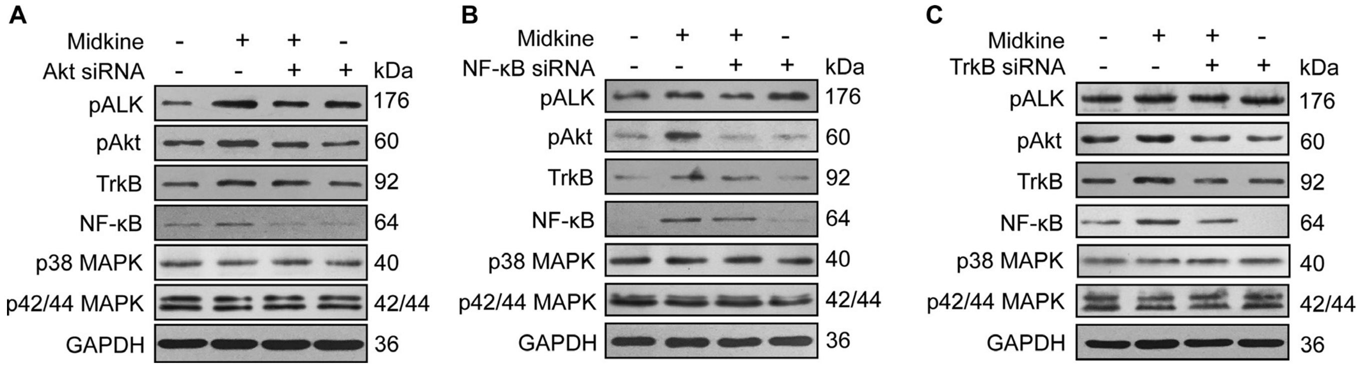

Supplementary Figure 4: PI3K/Akt/NF-κB/TrkB signaling participates in midkine-induced anoikis resistance of Hep3B cells. Expressions of signaling pathway-related proteins in suspension-cultured Hep3B cells with siRNA targeting Akt (**A**), NF-κB (**B**), and TrkB (**C**) treated with/without midkine (20 ng/mL) for 24 hours, detected by Western blotting.

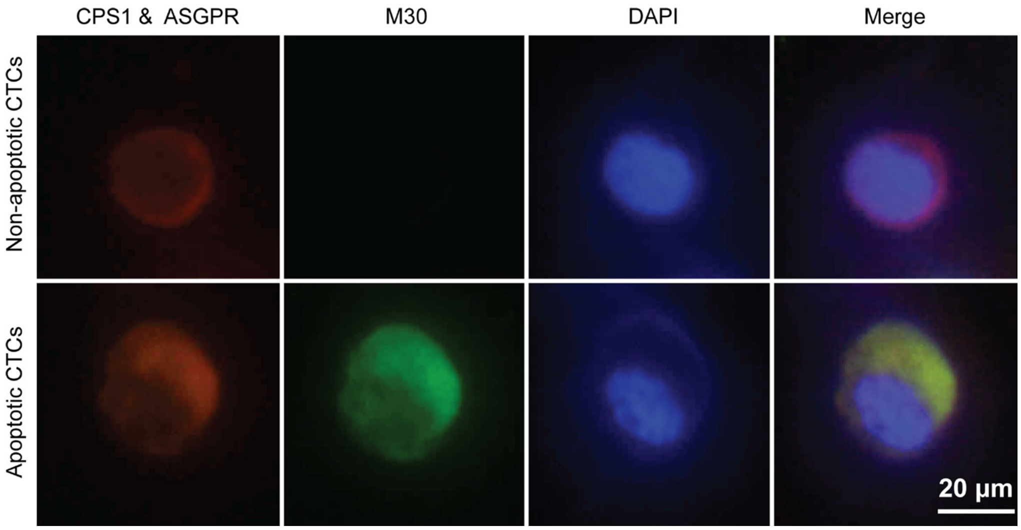

Supplementary Figure 5: Expression of M30 in circulating tumor cells (CTCs) from patients with hepatocellular carcinoma (HCC). Red: carbamoyl phosphate synthetase 1 (CPS1) and asialoglycoprotein receptor (ASGPR); Green: M30; Blue: 4′,6-diamidino-2-phenylindole (DAPI). Scale bar: 20 μm.

Supplementary Table 1: Correlations of midkine with clinicopathologic features in 341 patients with HCC

Variable	Total (n = 341)	Midkine-elevated (n = 238)	Midkine-normal (n = 103)	p
Age, ≤ 50/ > 50 years	165/176	110/128	55/48	0.223
Sex, male/female	281/60	193/45	88/15	0.333
Hepatitis, HBV/HCV/none	281/36/24	196/23/19	85/13/5	0.450
Serum AFP, ≤ 20/> 20 ng/mL	156/185	111/127	45/58	0.616
Liver cirrhosis, yes/no	287/54	204/34	83/20	0.233
BCLC stage, 0–A/B/C	248/49/44	196/20/22	52/29/22	< 0.001
Tumor size, ≤ 5.0/> 5.0 cm	134/207	91/147	43/60	0.542
Tumor number, single/multiple	255/86	179/59	76/27	0.781
TNM stage[a], I/II/III	95/175/71	66/112/60	29/63/11	0.006
Tumor encapsulation, yes/no	146/195	101/137	45/58	0.830
Vascular invasion[b], yes/no	184/157	138/100	46/57	0.023

AFP, alpha-fetoprotein; BCLC, Barcelona clinic liver cancer; HBV, hepatitis B virus; HCC: hepatocellular carcinoma; HCV, hepatitis C virus; TNM, tumor-node-metastasis.
[a]Sixth edition of International Union Against Cancer (UICC) TNM staging system (2002);
[b]Defined by findings on final pathological analysis.

Supplementary Table 2: Comparison of apoptotic CTCs in patients with HCC and midkine-elevated levels or midkine-normal levels

	Midkine-elevated (n = 58)	Midkine-normal (n = 44)	p
CTC count	19.5 ± 10.4	14.8 ± 7.1	0.012
Apoptotic CTCs, %	31.2 ± 17.1	69.6 ± 13.8	< 0.001
Apoptotic CTC count	6.2 ± 2.7	10.1 ± 3.7	0.000
